# Peer counseling for perinatal depression in low- and middle-income countries: A scoping review

**DOI:** 10.1017/gmh.2024.73

**Published:** 2024-10-18

**Authors:** Alexander Cuncannon, Kailyn Seitz, Aneel Singh Brar, Aliyah Dosani

**Affiliations:** 1 Faculty of Nursing, University of Calgary, Calgary, AB, Canada; 2 Owerko Centre, Alberta Children’s Hospital Research Institute, University of Calgary, Calgary, AB, Canada; 3 School of Nursing and Midwifery, Mount Royal University, Calgary, AB, Canada; 4 School of Public Health, University of Alberta, Edmonton, AB, Canada; 5 Mata Jai Kaur Maternal and Child Health Centre, Sri Ganganagar, India; 6 School of Anthropology and Museum Ethnography, University of Oxford, Oxford, UK; 7 Brain and Mind Institute, Aga Khan University, Nairobi, Kenya; 8 Addictions and Related Research Group, Sangath, Socorro, Goa, India; 9 Department of Community Health Sciences, Cumming School of Medicine, University of Calgary, Calgary, AB, Canada; 10 O’Brien Institute for Public Health, University of Calgary, Calgary, AB, Canada

**Keywords:** global health, peer counseling, perinatal depression, perinatal mental health, task sharing

## Abstract

Perinatal depression is associated with adverse maternal, newborn and child health outcomes. Treatment gaps and sociocultural factors contribute to its disproportionate burden in low- and middle-income countries (LMICs). Task-sharing approaches, such as peer counseling, have been developed to improve access to mental health services. We conducted a scoping review to map the current literature on peer counseling for perinatal women experiencing depression in LMICs. We searched CINAHL, MEDLINE, APA PsycINFO, Global Health and EMBASE for literature with no date limits. We included 73 records in our analysis, with most being systematic reviews and meta-analyses, randomized controlled trials and qualitative studies. Most studies were conducted in India and Pakistan and published from 2020 onward. The Thinking Healthy Program (THP) and its Peer-Delivered (THPP) adaptation were the most common interventions. Studies suggested effectiveness, feasibility, acceptability and transferability of peer counseling, particularly within the THPP, for perinatal depression. Studies indicated that local women, as peers and lay counselors, are preferred and effective implementation agents. Gaps in the evidence include those relating to understanding perinatal depression (e.g., contextual understandings of the etiology, comorbidity and heterogeneity and social conditions of psychosocial distress including long-term impacts on relationships and children’s development) and understanding and improving implementation. Further research on the adaptation, scaling up and integration of peer-delivered approaches with other approaches to improve impact are needed. There are also gaps in understanding the perspectives and experiences of peer counselors. Evidence gaps may stem from an emphasis on conventional public health approaches and measures derived from Western psychiatry, such as randomized controlled trials. There is relatively little research or implementation that prioritizes peer counselors in terms of understanding their perspectives and experiences (e.g., of professionalization), despite them being central to peer-delivered models. Task sharing has the potential to both empower peer counselors through mental health benefits and professional opportunities but also render peer counselors susceptible to vicarious exposure to traumatic stories and difficult situations amid limitations in available support. Better understanding counselors’ and perinatal women’s experiences can help decolonize the evidence base and improve implementation.

## Impact statements

Perinatal depression, which may be experienced by women during pregnancy to 1 year postpartum, can be debilitating for mothers and families. Perinatal women may be affected by functional and self-care impairments and suicidality, while infants and children may be affected by preterm birth, low birth weight and social and cognitive developmental delays. Treatment gaps and sociocultural factors contribute to a higher rate and burden of perinatal depression in low- and middle-income countries (LMICs). Task-sharing approaches, such as peer counseling, have been developed to improve women’s access to perinatal mental health care. This scoping review synthesizes 73 studies that examine peer counseling for perinatal depression in LMICs. Most studies were conducted from 2020 onward and in India and Pakistan. The Thinking Healthy Program (THP) and its Peer-Delivered (THPP) adaptation were the most frequently employed interventions. Studies indicated the effectiveness, feasibility, acceptability and transferability of peer counseling for perinatal depression. Studies highlighted perinatal women’s preference for local women as peer counselors, emphasizing their effectiveness and suitability as implementation agents. Task sharing has the potential to empower peer counselors through mental health benefits and professional opportunities, but it also poses risks, such as exposure to vicarious trauma and challenging situations with limited available support. Knowledge gaps included mixed evidence on the nuances of perinatal depression (e.g., multifactorial causes, co-occurrence with other mental disorders and sociocultural influences) as well as limited evidence on the perspectives and experiences of peer counselors. This scoping review may inform future research, including adaptation, scaling up and integration of peer-delivered approaches with other approaches to improve impact. Given their pivotal roles in peer-delivered models, research and implementation must prioritize understanding the perspectives and experiences of peer counselors. A deeper understanding of counselors’ and perinatal women’s experiences can contribute to decolonizing the evidence base and enhancing implementation strategies.

## Introduction

Depression is a leading cause of morbidity and mortality worldwide. In addition to individual, family and community-level impacts, depression has a significant economic burden (Arias et al., 2022). Perinatal depression, which may be experienced by women during pregnancy to 1 year postpartum, can be debilitating with respect to feelings of sadness and hopelessness, fatigue and anxiety and changes in appetite and sleep (Letourneau et al., 2017; Ransing et al., 2020; Roddy Mitchell et al., 2023). Women who experience perinatal depression may be affected by negative outcomes, including functional and self-care impairments as well as suicidality, while infants and children may be affected by preterm birth, low birth weight and social and cognitive developmental delays (Grote et al., 2010; Goodman et al., 2011; Premji et al., 2015; Lebel et al., 2016; Madigan et al., 2018). Women living in low- and middle-income countries (LMICs) are rendered particularly vulnerable as they experience higher rates of perinatal depression and greater barriers to accessing mental health care (Gavin et al., 2005; Grote et al., 2010; Fisher et al., 2012; Upadhyay et al., 2017). A recent meta-analysis found that one in four women living in low-resource countries reported experiencing perinatal depression, which may go unidentified amid gaps in mental health support and care systems (Roddy Mitchell et al., 2023). This disparity may be influenced by limited social support, poverty and sociocultural factors, such as the stigma associated with mental health disorders and various barriers that prevent women from seeking care (Patel et al., 1999; Raman et al., 2014; Goyal et al., 2020).

Perinatal depression can be effectively managed with psychological treatment, which provides benefits for both parenting and child development (Letourneau et al., 2017). In LMICs, however, a treatment gap between the need and availability of mental health services persists and is estimated to be greater than 90% (Patel et al., 2010, 2011; Thornicroft et al., 2017; Ransing et al., 2020). To increase the availability and accessibility of mental health services in low-resource settings and underserved communities within LMICs, task-sharing approaches (i.e., training and supporting nonspecialist community health care workers [CHWS] or lay community members to provide health care services) have been developed (Clarke et al., 2013).

Several psychosocial interventions have been designed specifically for the perinatal period and have further been adapted for peer-delivered approaches. In 2015, the World Health Organization published the Thinking Healthy Program (THP), a manual for treating perinatal depression in low-resource settings (World Health Organization, 2015). The Thinking Healthy manual guides the integration of cognitive-behavioral therapy (CBT)-based strategies into the work of nonspecialist providers such as community health workers (Singla et al., 2014; Atif et al., 2015; World Health Organization, 2015). To further address human resource gaps, the THP was adapted to the THP Peer-Delivered (THPP), whereby peers and lay community members counsel women experiencing perinatal depression (Singla et al., 2014; Atif et al., 2015). Peer and lay counselors may be generally, although not universally, differentiated from CHWs as peers may be members of local communities, share some mutual life and health experiences with the women and families they engage with, and have more focused scopes of intervention (Singla et al., 2014).

Although the THP and THPP have been extensively researched and provide much of the current evidence base on task-sharing psychosocial interventions for the management of perinatal depression, the evidence is broadening and incorporating more peer counseling approaches and therefore warrants synthesis. A recent systematic review and meta-analysis of only randomized controlled trials (RCTs) of task-sharing interventions for common mental disorders (CMDs) found tentative evidence of effectiveness in low-resource countries, although considerable heterogeneity contributed to uncertainty about long-term effects (Prina et al., 2023). Although CMDs may have shared etiology and phenomenology, depression is often a target of intervention given its associated mortality and morbidity. Furthermore, while extensive research has suggested clinical and cost-effectiveness of THP and THPP, there is less evidence on cultural transferability, implementation strategies, scale-up and sustainability across settings and populations (Rahman et al., 2021; Prina et al., 2023). To inform future research on the broader evidence base of perinatal mental health in LMICs, we conducted a scoping review to map the current literature on peer counseling for perinatal women experiencing depression in LMICs.

## Methods

Scoping reviews explore the breadth of and summarize available research evidence (Arksey and O’Malley, 2005; Tricco et al., 2018; Peters et al., 2020). Our aim was to characterize available evidence and identify knowledge gaps to inform global health research in the context of perinatal mental health, particularly in low-resource settings. Our research question was: how are peer and lay counselors bridging gaps in perinatal mental health services in LMICs? We conducted our review using Arksey and O’Malley’s (2005) framework of identifying the research question, identifying relevant studies, selecting studies, charting data and collating and reporting results. We present our results in accordance with the PRISMA Extension for Scoping Reviews reporting guideline (Tricco et al., 2018).

### Eligibility criteria

Within the literature on task-sharing psychosocial interventions delivered by CHWs, we focused on literature that examined interventions delivered entirely or in part by peer or lay counselors (i.e., peer counseling) for perinatal depression. We defined peer and lay counselors as nonspecialist community members who had no formal mental health training prior to professional training (Connolly et al., 2021). We included studies with a population focus of women in the perinatal period residing in World Bank-classified low-income and LMICs. We defined the perinatal period from conception up until 12 months postpartum, although longitudinal studies that continued beyond this period were also eligible. Studies of any design were eligible for inclusion. We excluded studies that examined programs delivered in upper-middle-income and high-income countries as classified by the World Bank. We also excluded non-English-language literature.

### Information sources and search

A health sciences librarian (CM) at Mount Royal University developed our search strategy in collaboration with our team based on discussions about concepts related to population, intervention and outcome. Complete search strategies for each database are available in Supplementary Material S1. We searched CINAHL Plus with Full Text, MEDLINE and APA PsycINFO *via* EBSCOhost as well as Global Health and EMBASE *via* OVID from inception to July 7, 2023. No restrictions on publication date were applied to searches. Additional records were identified through citation searching and consultation with team members.

### Selection of sources of evidence, and data charting process and items

We imported and screened records in Covidence, a collaborative web-based platform that streamlines the production of systematic and other literature reviews (Veritas Health Innovation, Melbourne, Australia). Two reviewers (KS, AC) screened the title and abstract associated with each record. Percent agreement at title and abstract screenings was 87%. Conflicts at this level were resolved by a third team member (AD). Records that proceeded to full-text screening were read in full and screened. Percent agreement at full-text screening was 84%. Two reviewers (KS, AC) extracted data into a standardized template in Covidence. For each record, information including title, author, country or countries included, setting, funding sources, conflicts of interest, methods, aims and design were extracted. For observational and interventional studies, information including start and end dates, participant characteristics, assessment of perinatal depression, intervention classification, recruitment and training of lay counselors, program format, content and duration, comparator group, data collection methods, effects on maternal mental health, other findings and key interpretations were extracted. For reviews, information on type of review, number of studies included, number of studies included applicable to this scoping review, findings and key interpretations were extracted. For protocols, information on type of study, registration, status, objectives and participants were extracted. Two reviewers (KS, AC) used collaborative brainstorming software to identify key themes, trends and knowledge gaps noted throughout the screening and analysis process. We met as a team to discuss the findings and how themes, knowledge gaps and implications would be communicated in our discussion.

## Findings

**Figure 1.**
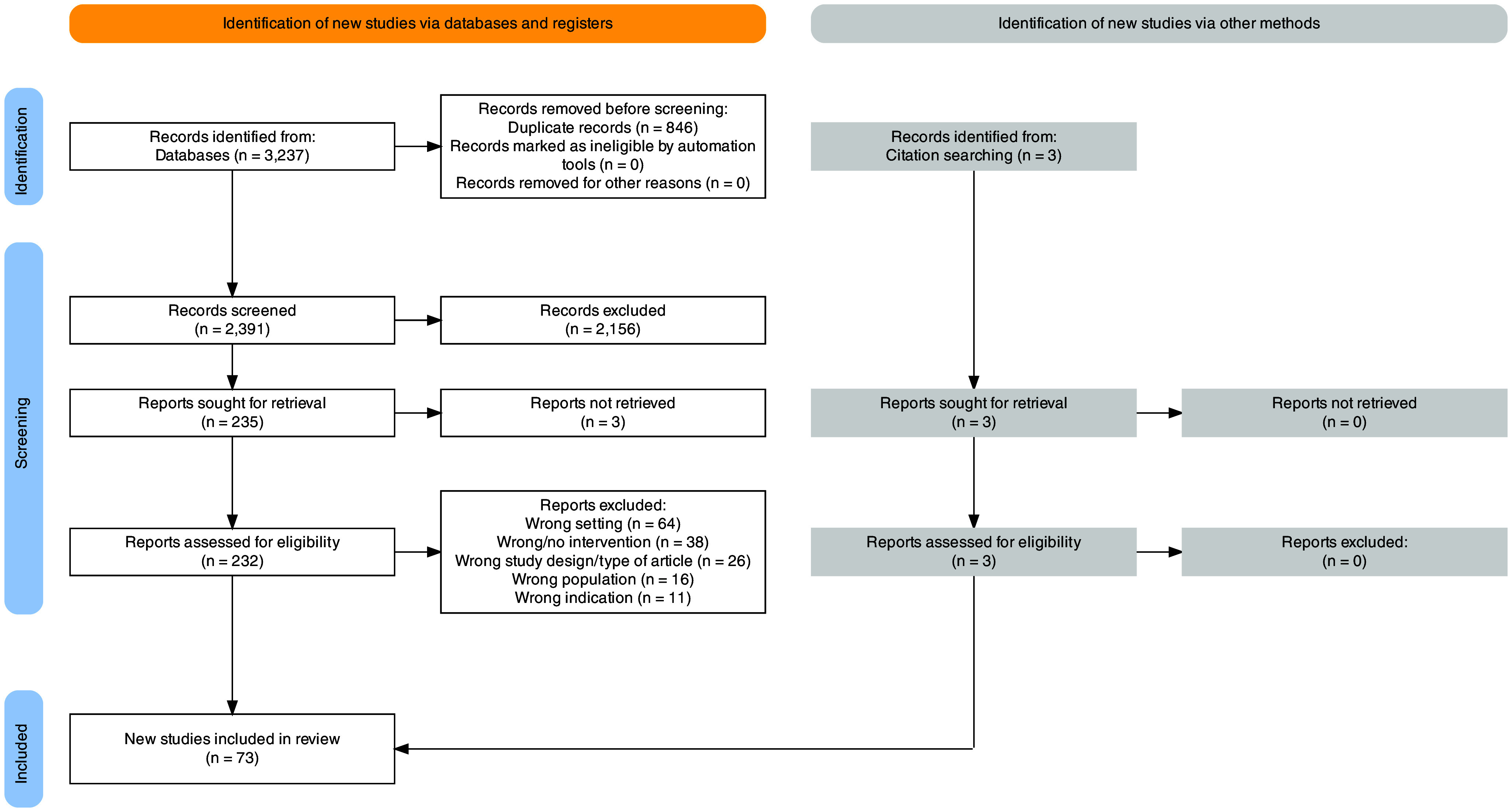
PRISMA flow diagram.

As shown in the PRISMA flow diagram (Figure 1; Haddaway et al., 2022), the search strategy identified 3,237 records, of which 846 records were duplicates. At the title/abstract screening stage, 2,156 records were excluded. At the full-text screening stage, we sought 235 records for retrieval and assessed 232 records for eligibility, as three records were not accessible through journal websites, library collections or interlibrary loan. Of these records, 162 were excluded for reasons including impertinent setting, intervention, design, sample indication or non-English-language. Three records were identified from citation searching and included in the review after assessing eligibility at the full-text screening stage.

**Figure 2.**
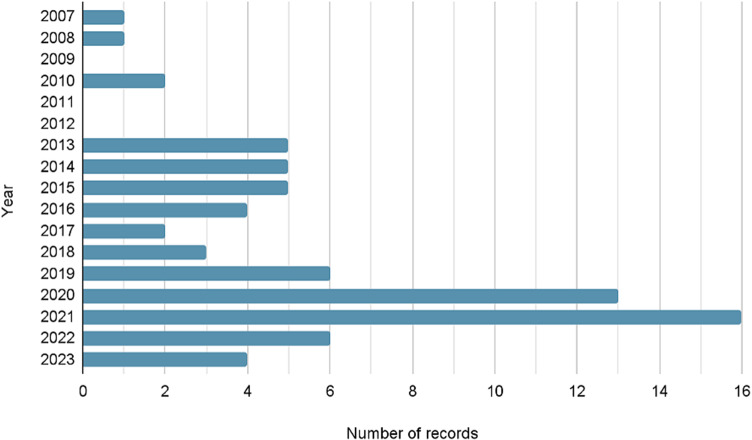
Publication years of included records.

The 73 included records were published between 2007 and 2023, and more than half were published since 2020 (Figure 2). The most frequent studies were RCTs (13), qualitative studies (13), systematic reviews and meta-analyses (12) and cohort studies (7). Twelve systematic reviews and meta-analyses and one meta-analysis were included. Then, 43 included studies directly examined task-sharing perinatal mental health interventions, and most (67%) studies occurred in Pakistan (27) and India (11), followed by Kenya (6) and Bangladesh (3). THP and THPP were the most common interventions, in 63% of these studies. The types of included records, an overview of included systematic reviews, geographies of included interventional studies, primary programs of included interventional studies and an overview of articles examining THPP are shown in Supplementary Material S2.

### Systematic reviews and meta-analyses

The 13 included systematic reviews and meta-analyses span overlapping samples, such as Rahman et al.’s (2008) cluster RCT. Major themes identified include the value of contextual and culturally adaptation, screening and awareness (Padmanathan and de Silva, 2013; Chowdhary et al., 2014; Munodawafa et al., 2018; Fang et al., 2022; Zhu et al., 2022). Several systematic reviews highlighted issues related to the heterogeneity and quality of evidence (Dennis and Dowswell, 2013; van Ginneken et al., 2013; van Ginneken et al., 2021; Prina et al., 2023). Collectively, these systematic reviews present collective evidence that supports task-sharing approaches to bridge gaps in perinatal mental health care in LMICs (Clarke et al., 2013; Rahman et al., 2013; Chowdhary et al., 2014; Fang et al., 2022).

### THP and THPP

Eleven included studies examined task-sharing programs focused on the THP. In a qualitative study that included interviews and focus group discussions, Singla et al. (2014) explored desired characteristics of and considerations regarding peers who could deliver the THP; this formative research shaped the development and implementation of the THPP. In a 5-year review of the THP, Rahman et al. (2021) described lessons learned from THP across India, Pakistan, Vietnam and Kenya, including its effectiveness to integrate with existing maternal-child health programs as well as task-sharing and cascaded approaches, cultural adaptability and opportunities to use technology for training, supervision, delivery and scale-up.

Sixteen included studies that examined task-sharing programs focused on the THPP (Supplementary Table S5). Most included articles that examined THPP stemmed from the concurrent South Asian Hub for Advocacy, Research and Education for mental health (SHARE) Rawalpindi, Pakistan and Goa, India RCTs (Sikander et al., 2015). These trials, which compared THPP and enhanced usual care (EUC) to EUC alone, positioned THPP as clinically and cost-effective as well as scalable (Fuhr et al., 2019; Sikander et al., 2019).

#### Embedded studies

Numerous included studies were embedded within the SHARE trials. Regarding strength of implementation in SHARE Pakistan, peer volunteers demonstrated good scores on an implementation index (constructed from four indicators: THPP session count and duration, peer competency checklist scores and monthly supervision session attendance); however, there was no significant association between implementation indices and PHQ-9 scores, and it was posited that other constructs (e.g., individual participant-level data) may have been underlying the association between the THPP intervention and PHQ-9 scores (Ahmad et al., 2020). In a qualitative study embedded within SHARE Pakistan, personal gains, family and community endorsement, and good training and supervision were identified during interviews and focus group discussions as facilitators of peer volunteers’ motivation, while reluctance to engage among women and families were identified as barriers (Atif et al., 2016). Atif and colleagues identified high levels of need, desirable peer volunteer characteristics and linkages with the local primary health care system as facilitators and mental health stigma and sociocultural factors as barriers, to community acceptability (Atif et al., 2016). In an adaptation and feasibility study within both trials, during focus groups and field testing, most mothers reported some improvement, including improved coping and motivation to act and spend more quality time with their children, and peers were generally perceived to have good sociocultural understanding and relatability (Atif et al., 2017). Peers framed their roles as enabling them to learn new skills and enhance employment opportunities, although there was an expressed desire for monetary incentives for peers in Goa, in contrast to rural Rawalpindi where status and goodwill were perceived as sufficient (Atif et al., 2017). These embedded studies provide collective evidence on THPP implementation and sustainability in Pakistan and India.

#### Longitudinal analysis

Several included articles presented longitudinal follow-up analysis of the SHARE trials. In a 3-year follow-up to SHARE Pakistan, there were no significant outcome differences in PHQ-9 and Strengths and Difficulties Questionnaire, Total Difficulties (SDQ-TD) scores observed between intervention and EUC arms (Maselko et al., 2020). Over these 3 years, about 40% of mothers did not complete the entire THPP+ intervention and peer volunteers’ competence levels declined over time and especially after 12 months’ time (Maselko et al., 2020). In an evaluation 5 years after its initiation, 70% of peer volunteers in the original cohort continued to participate and all achieved satisfactory competence (Atif et al., 2019a). In this 5-year report, altruism, enhanced social standing, improved personal mental health and the possibility for other employment opportunities were identified as factors contributing to peers’ sustained motivation, while demotivation associated with uncertainty about the program’s future, preference for financial incentives and reluctance to ongoing care from some families were identified as challenges (Atif et al., 2019a). These studies and further longitudinal research provide insight on sustainability and scale-up of THPP.

#### Secondary analysis

Several included articles were secondary analyses. In a multiple mediation analysis of the SHARE trials, participant-reported activation and social support, but not mother–child attachment, were found to mediate the effects of THPP on depression outcomes at 6 months (Singla et al., 2021). In a pooled analysis of the trials, participants in the intervention arm had lower PHQ-9 scores and higher odds of remission, and the intervention effect was greater among women who were primiparous and who had shorter depression chronicity (Vanobberghen et al., 2020). In a secondary analysis of THP, Waqas et al. (2022) presented a prognostic model for predicting remission of perinatal depression among women and found that participation in THP, living in a joint family system and with infants’ grandmothers, higher socioeconomic status, lower severity scores of core emotional symptoms, insomnia and atypical symptoms (e.g., hypersomnia, hyperphagia and weight gain) were associated with a better prognosis. Altogether, secondary analysis reinforces the findings of initial studies and illuminates linkages over time.

#### Qualitative analysis

Two included ethnographic studies examined the SHARE India trial. Based on fieldwork conducted during the trial, Leocata et al. (2021b) identified three primary themes in peer counselors’ (sakhis’) experiences of caregiving: caregiving as a relational exchange and reciprocated process, memories of care and moral resonance and awareness surrounding disengagement and removal of care. The sakhis’ perceptions of care in the present and in the past, during their own pregnancies, were highlighted. In an exploration and contrasting of material technologies within the SHARE India trial as well as during fieldwork in Kenya, Leocata and colleagues (2021a) revealed how material technologies of care can both facilitate relational engagement and constrain care. They highlighted tensions between intervention materials promoting sakhis’ experience and expertise, and overarching manualization and protocolization (Leocata et al., 2021a). Qualitative analyses of THPP center perspectives of peer-counselors amid dynamic social, cultural and political forces.

### Factors influencing peer counseling

Factors influencing peer counseling relationships and interventions were identified in included studies. In a systematic review and metasynthesis, Zhu and colleagues (2022) highlighted close consideration of women’s social determinants of health, psychoeducation to address stigma and therapeutic relationships as facilitators. Recruitment and training of CHWs and peer counselors was heterogeneous across included studies. Several authors underscored the value of counselors sharing similar cultural and social contexts as well as experiences, particularly motherhood and perinatal depression, with the women they cared for (Rahman et al., 2013; Singla et al., 2014; Atif et al., 2016; Munodawafa et al., 2018; Mohsin et al., 2021; Ng’oma et al., 2022). Examples of cultural adaptations included modification of THP content and small group delivery in Vietnam (Fisher et al., 2014), review of THPP illustrations and adaptation of language, stories and idioms in Malawi (Ng’oma et al., 2022), and local development and use of screening tools in Pakistan (Ali et al., 2010). Aspects of shared experiences between perinatal women and peer counselors as well as cultural adaptation were illustrated as important ethical responsibilities and for effective engagement of women and families.

Structural social determinants of maternal mental health were explored. Rahman (2007), Gajaria and Ravindran (2018), and Baumgartner and colleagues (2021) suggested that social, economic and cultural factors (e.g., poverty, illiteracy, stigma and barriers to accessing health care) that may shape depressive symptoms as well as women’s perceptions and abilities to seek and engage with mental health supports be considered in the adaptation of content and delivery. THP was framed as adaptable to address factors including poverty, social insecurity and relational issues (Rahman et al., 2008; Malqvist et al., 2016). Family and community engagement were framed as strategic in building capacity and supporting long-term behavior change (Rahman et al., 2013; Singla et al., 2014). Davies and colleagues (2019) and Prina and colleagues (2023) underscored the need to further address root determinants of health, especially social support networks, in interventions going forward. Altogether, these studies suggest that the social support inherent in peer counseling augments the effectiveness of psychological interventions.

Peer counselors’ roles spanning screening, detection, assessment and referral were illustrated. Innovative mobile health strategies included integration of passive sensing and behavior change strategies and digital platform and cascaded training (Rahman et al., 2019; Lakshminarayanan et al., 2020; Byanjankar et al., 2021). Aspects of workload and burnout (Rahman, 2007; Rahman et al., 2008; Zafar et al., 2014) as well as altruism, intrinsic motivation, remuneration and employment prospects were also explored (Singla et al., 2014; Atif et al., 2017, 2019b).

### Child development

Maselko and colleagues (2015) evaluated the impact of the THP RCT on child development at 7 years post-intervention and found no difference in children’s cognitive, socioemotional and physical development between the trial arms. These authors suggested several explanations may have contributed to this, including changes in children’s developmental trajectories and intervention effects being latent at age 7. With that said, a correlation was noted between third-trimester depression and children’s SDQ-TD and SCAS anxiety scores independent of mothers’ current depressive symptoms, suggesting that third-trimester depression may uniquely shape children’s socioemotional development (Maselko et al., 2015). Maselko and colleagues (2020) then examined the effects of THPP on maternal depression and child development outcomes at 3 years of age in a cluster RCT and found no significant outcome differences between treatment and EUC arms. They suggested that treatment effects may have been masked by low symptom severity and high remission rates across both arms, and that the intervention may not have been frequent or intensive enough to modulate the effect of perinatal depression on child development at age 3.

### Cultural and technological adaptation

Several studies reported on cultural and technological adaptation. In Tomlinson and colleagues’ (2020) exploratory feasibility study of an adaptation of THP in Afghanistan, THP was considered feasible to the Afghan context with a reduced number of sessions. Ng’oma and colleagues (2022) conducted a qualitative study to examine the cultural appropriateness of THPP in Lilongwe, Malawi and found that THPP was considered appropriate for the target population after adaptation including translation, illustration redesign, integration of culturally responsive stories and idioms and modified session frequency. Atif and colleagues (2022) conducted a usability testing study of a technology-assisted and THPP in Rawalpindi that positioned peers and the app as ‘co-therapists.’ Khan and colleagues’ (2017) RCT of the ‘Happy Mother, Healthy Child in Ten Steps’ intervention was conducted in a post-conflict context of humanitarian emergency and displacement in the Swat valley. The authors emphasized ‘child’ aspects of the intervention while avoiding direct reference to mental illness and reported that delivery and uptake in the intervention arm were effective (Khan et al., 2017).

### Lay and peer counselors’ experiences of caregiving and professional socialization

A handful of articles explored peer counselors’ first-hand experiences. In their ethnographic study, Leocata and colleagues (2021b) described how sakhis (counselors) shared challenges with ending peer-counseling care, which they perceived as needing to continue, during the THPP trial. The sakhis analogized this to care that they felt was lacking during their own pregnancies. In this vein, THPP was framed as a moral engagement and the caregiver experience was highlighted as understudied, presenting concerns with respect to intervention delivery and sustainability (Leocata et al., 2021b). In another ethnographic study, Leocata and colleagues (2021a) problematized RCTs, like those conducted for THPP, being regarded as among the highest levels of evidence. Several included articles described elements of professional socialization and motivation. Sikander and colleagues (2019) illustrated peer roles as a form of social investment as peers may receive in-kind returns from their communities, while Atif and colleagues (2019a) envisioned peers’ career paths and continuing professional development.

## Discussion

This scoping review maps the available research evidence on peer counseling interventions for women experiencing perinatal depression in LMICs. Recent systematic reviews indicate that lay health workers’ and other community members’ engagement in mental health interventions may benefit individuals who experience mental disorders and distress, but that more evidence is needed (van Ginneken et al., 2021; Prina et al., 2023). Most interventions within the included studies in this review were based on or derived from CBT, similar to Prina and colleagues (2023) recent systematic review of RCTs. Prina and colleagues (2023) suggested that the evidence base should be broadened to encompass a variety of prevention settings and approaches to address the social determinants of maternal mental health.

The findings of this scoping review indicate a predominance of and evolution of THP and THPP in the literature. Evidence suggests the effectiveness, feasibility, acceptability and transferability of the THP and THPP (Vanobberghen et al., 2020; Rahman et al., 2021). Furthermore, peer-delivered approaches show effectiveness as frontline interventions in stepped-care models where screening, early detection, delivery, and referral are integrated into existing health care systems and communities (Vanobberghen et al., 2020).

One of the most salient findings across studies is local women’s and other stakeholders’ preference for peers and laywomen as counselors and the primary agents of implementation (Singla et al., 2014; Sikander et al., 2019; Leocata et al., 2021a, 2021b). This preference may be grounded in practicality (i.e., the availability of human resources for mental health), shared experiences of peers and their perinatal patients and efficacy of counseling relationships. Peer counseling approaches encompass the core components identified by McNab and colleagues (2022) in a landscape analysis as contributing to the success of perinatal mental health interventions at the community level including task-sharing, stepped care, talk therapy and contextual assessment. Further, peer counseling broadens and shapes women’s social support in uniquely relational ways (Singla et al., 2014; Atif et al., 2019a; Leocata et al., 2021a, 2021b). However, in their systematic review of RCTs, Prina and colleagues (2023) found that few studies reported on how interventions affected women’s social support. Leocata and colleagues (2021a, 2021b) problematized the positioning of RCTs with a quantitative and data-driven focus. Given the importance of peers and lay women to the implementation, efficacy and potential for scale-up of task-sharing approaches, there is relatively little research examining their experiences and further integrating these lessons. Importantly, adaptation and implementation research can transcend some of the limitations in conventional epidemiological measures and methods.

### Parent–child relationships, neurobiology and child development

While maternal mental health outcomes are at the forefront of THP and THPP, perinatal depression has far-reaching effects on child development (Goodman et al., 2011; Kingston and Tough, 2014). Parenting and child development outcomes were examined in relatively few included articles. In their 7-year longitudinal follow-up to the THP RCT in Pakistan, Maselko and colleagues (2015) highlighted challenges in exploring the differential impact of chronic *versus* recurrent depressive episodes on child developmental outcomes. In Singla and colleagues’ (2021) multiple mediation analysis of THPP-India and THPP-Pakistan, although no effect on mother–child-reported attachment was found, this may have been related to measurement and lack of emphasis on attachment in that particular delivery. Further research with more attachment-focused assessment methods and independent observation may better explain how these programs shape attachment. Amid contexts of gender inequality and gender-based violence and adversity, further research that incorporates neurobiology lenses may also offer insights on biological embedding of stress. In an examination of hair-derived biomarkers of hypothalamic–pituitary–adrenal axis function in participants of the SHARE Pakistan trial (Sikander et al., 2019), Baranov and colleagues (2022) reported that mothers in the intervention group had lower cortisol, lower cortisone and higher dehydroepiandrosterone (DHEA) levels than mothers in the EUC group and moreover that infants of these mothers had higher DHEA levels. This suggests that THP and THPP+ may buffer stress-related responses intergenerationally. Work remains to consider intervention effects at relational, epigenetic, and neurobiological levels, and to examine longer-term implementations and cohorts for effects on parent–child relationships and child development.

### Mental health comorbidity and heterogeneity

The nuanced dimensions of perinatal psychosocial distress hold research and practice implications. Perinatal depression has heterogeneous presentations and dynamic trajectories (Waqas et al., 2023). To work toward more tailored approaches to intervention, Waqas and Rahman (2021) identified four clinical phenotypes of perinatal depression (mixed anxiety, somatic, mild, and atypical), all of which responded well to THP and suggested the adoption of a transdiagnostic framework and network perspective in future research. Moreover, women’s well-being is affected by many factors of psychosocial health. As examined in cohorts of pregnant Pakistani women, depression and anxiety are often comorbid and of multifactorial etiology, and these symptoms may have predictive value of preterm birth (Premji et al., 2020; Lalani et al., 2021, 2023). In a process evaluation in Rawalpindi, nonspecialists effectively delivered a CBT-based *Happy Mother Healthy Baby* intervention to perinatal women experiencing anxiety but not depression according to the Structured Clinical Interview for the Diagnostic and Statistical Manual of Mental Disorders (SCID) (Atif et al., 2020, 2023). Future research could include examination of how interventions affect comorbid perinatal depression and anxiety, suicidality, other comorbidities including substance use and experiences of gender-based violence. Improved understandings of heterogeneity in presentations and trajectories of perinatal depression can help tailor peer counseling.

### Leveraging and integrating of peer counseling models

McNab and colleagues (2022) underscored value in integrating interventions across health sectors (e.g., maternal-newborn-child, nutrition, and HIV care initiatives), beyond health (e.g., to education, gender, and economic empowerment initiatives) and across primary health care. Peer counseling models may be leveraged to integrate more broadly with other psychosocial intervention modalities for mental health given risk factors that include social determinants of maternal mental health (e.g., economic and gender inequality, and gender-based violence and stillbirth) (McNab et al., 2022). For instance, women who experience perinatal depression may also experience gender-based violence and women’s partners’ mental health may be a source of distress, such as in the context of substance use disorders. THP does not explicitly address either of these aspects, so it is worth exploring how THP and THPP may be integrated with initiatives that target and reduce gender-based violence and adversity and promote mental health from additional dimensions such as paternal mental health (Sattar et al., 2023).

### Local adaptation, development and scale-up

Much of the evidence for THP and THPP is derived from Pakistan and India. As many included articles suggested, local adaptation depends on measuring depression and outcomes in contextually appropriate and ethical ways, understanding local idioms of distress and culture-bound syndromes, understanding local risk factors (e.g., gender preference) and other aspects of local culture, context, politics and power (Farmer, 2004; Perera et al., 2020). Although research on THPP has included cultural and local adaptation and scale-up, measures derived from Western psychiatry (e.g., PHQ-9, Edinburgh Postnatal Depression Scale, and SCID) persist, and further work is needed to develop more locally and culturally applicable and responsive measures (Dosani et al., 2022).

Peer counseling models have the potential to be both locally responsive and scalable, but large-scale implementation has yet to occur. Dynamics may shift during scale-up and further formative research is required to adapt interventions to scale-up implementation as well as to examine issues of voltage drop and program drift over time. Opportunities for technology, mobile health, and intersectoral partnerships exist to address these complexities in addition to concerns regarding privacy, use of data, and disparities in access, especially as technology evolves (Atif et al., 2019a; Rahman et al., 2019; Dosani et al., 2020).

### Peer and lay counselors’ experiences of professionalization and scaling up

Singla and colleagues’ (2014) formative work on counselor profiles and relationships, Leocata and colleagues’ (2021a, 2021b) ethnographic work on sakhis’ during- and end-of-trial experiences and Wood and colleagues’ (2021) work on lay health workers’ perceived barriers and facilitators should inform cultural and local adaptation as well as scale-up. Atif and colleagues (2019a) illustrated some potential mental health and livelihood benefits for counselors, but further research is needed to work toward more thorough accounts and understandings of counselors’ experiences in all aspects of mental health interventions, from recruitment and training to scale-up to post-intervention life course. Further research could explore how counselors develop new knowledge, skills, and attitudes; build on their past and present experiences for future opportunities; as well as explore the experiences of perinatal women who are peer counseled in sharing their lived/living experience of transitioning to become counselors themselves. Many lessons learned may be transferable to the broader evidence base on perinatal mental health.

Health inequities stem from power imbalances (Farmer, 2004; Gore and Parker, 2019), and increased perspectives from peer counselors could help to address these power imbalances and further decolonize the evidence base. Prioritizing the local voices and experiences of counselors may contribute to the decolonization of global mental health (Bemme and D’souza, 2014; Mills, 2014). Peer counseling is positioned as an approach that mobilizes and shares power with community members, privileges lived/living experience, and challenges Western concepts of psychiatry. In their caregiving and counseling, non-professional counselors may experience improved mental health and well-being, employment prospects, and self-empowerment and transformation. Conversely, non-professional counselors may also be exposed to possible harms and ethical implications involved in ending care as well as being exposed to suicidality, gender-based violence, and other traumatic stories, especially if supports are limited compared to those available for professionals. Understanding counselors’ experiences through innovative, creative, interdisciplinary, and qualitative methods such as ethnography and photovoice is crucial for success of scale-up of interventions. Interdisciplinary work may encompass involving ethnographers, epidemiologists, psychiatrists, nurses and others, all while foregrounding those on the front lines.

### Limitations

This scoping review is limited by several factors. We acknowledge limitations with the scoping review methodology, including heterogeneity leading to breadth over depth of content as well as a lack of quality assessment of included records. Our scoping review also includes an overarching focus on THP and THPP, reflecting their relative predominance in the existing evidence base. Although we included several databases, we included only English-language literature and may have missed literature published in languages of LMICs as well as more interdisciplinary research, for example, economic development literature such as that by Baranov and colleagues (2020). Furthermore, we did not specifically seek gray literature for inclusion because our aim was to map the global mental health research evidence base on peer counseling approaches to perinatal depression in LMICs. As highlighted by Prina and colleagues (2023), evidence on perinatal mental health in LMICs is comparatively sparse, which reflects disparities in research capacity and underscores a need to contribute to research agendas to address these inequities.

## Conclusions

Peer counseling for perinatal depression is positioned to improve the health and lives of mothers, children and families by addressing treatment gaps, engaging community members, and adapting and attuning interventions to local and cultural contexts in LMICs. Gaps in the evidence include those relating to understanding perinatal depression, improving implementation, and understanding and integrating the experiences of peer counselors. There is a need to address these gaps using interdisciplinary approaches and prioritizing an understanding of local context and peer counselor experiences and perspectives. Better understanding and prioritizing counselors’ and perinatal women’s experiences and voices can help decolonize the evidence base and improve the scale-up and sustainability of interventions.

## Supporting information

Cuncannon et al. supplementary material 1 Cuncannon et al. supplementary material

Cuncannon et al. supplementary material 2 Cuncannon et al. supplementary material

## Data Availability

Search strategies are available in Supplementary Material S1. The data that support the findings are available in Supplementary Material S2.

## Supplementary material

The supplementary material for this article can be found at http://doi.org/10.1017/gmh.2024.73.
