# Supplementary material for: Peer counseling for perinatal depression in low- and middle-income countries: A scoping review
Source: Glob Ment Health (Camb). 2024 Oct 18;11:e85. doi: 10.1017/gmh.2024.73 (PMC11504931; doi:10.1017/gmh.2024.73)
Supplement: Cuncannon et al. supplementary material 1 — Cuncannon et al. supplementary material [file S2054425124000736sup001.docx]

**CINAHL Plus with Full Text (via EBSCOhost)**

| **Population** | **Headings**  (MH "Pregnancy") OR (MH "Childbirth+") OR (MH "Pregnancy, High Risk") OR (MH "Pregnancy, Prolonged") OR (MH "Pregnancy, Multiple") OR (MH "Pregnancy, Unplanned") OR (MH "Pregnancy, Unwanted") OR (MH "Maternal Health Services") OR (MH "Maternal-Child Health") OR (MH "Postnatal Care") OR (MH "Postnatal Period") OR (MH "Puerperium") OR (MH "Prenatal Care") OR  (MH "Mothers+") |
| --- | --- |
|  | **Keywords**  pregnan* OR perinat* OR prenat* OR "post-partum" OR "post partum" OR postpartum OR antenat* OR mother* OR puerper* |
| **Intervention** | **Headings**  (MH "Peer Group") OR (MH "Community Health Workers") OR (MH "Volunteer Workers") |
|  | **Keywords**  "community health worker*" OR "community health agent*" OR "lay health worker*" OR "lay counsel*" OR "lay-counsel*" OR "alternative health worker*" OR "task shifting" OR "task sharing" OR "lay-delivered" OR "nonprofessional" OR "non-professional" OR "peer deliver*" OR "peer-deliver*" OR "peer volunteer*" OR "Thinking Healthy Program*" OR "scaling up" OR "scaling-up" OR "lady health worker*" OR "lady health visitor*" OR "friendship bench*" OR "barefoot therapist*" OR "barefoot doctor*" OR "community-based reproductive health agent*" OR "health extension worker*"​​ OR "accredited social health activist*" OR nonspecialist OR "non-specialist" |
| **Outcome** | **Headings**  (MH "Depression, Postpartum") OR (MH "Postpartum Psychosis") OR  (MH "Mental Health") OR (MH "Mental Disorders") OR (MH "Neurotic Disorders+") OR (MH "Pregnancy Complications, Psychiatric") OR  (MH "Psychotic Disorders") OR (MH "Adjustment Disorders+") |
|  | **Keywords**  "mental health" OR "mental disorder*" OR depress* OR "perinatal depression" OR "postpartum depression" OR "post-partum depression" OR distress* OR psych* N2 distress* OR anxi* OR mood OR nervous* OR sad* OR uneas* OR hopeless* OR worthless* OR "baby blue*" OR tens* OR fatigue* OR tired* OR nervous* OR suicid* OR agitat* OR somati* OR somati* N2 present* OR somati* N2 symptom* OR appetite OR "mental wellness" OR sleep OR "stress reduction" OR "emotional support" |

**MEDLINE (via EBSCOhost)**

| **Population** | **MeSH**  (MH "Pregnancy+") OR (MH "Maternal Health Services+") OR (MH "Mothers+) OR (MH "Maternal Health") OR (MH "Postpartum Period") |
| --- | --- |
|  | **Keywords**  pregnan* OR perinat* OR prenat* OR "post-partum" OR "post partum" OR postpartum OR antenat* OR mother* OR puerper* |
| **Intervention** | **MeSH**  (MH "Peer Group") OR (MH "Community Health Workers") OR (MH "Volunteers") |
|  | **Keywords**  "community health worker*" OR "community health agent*" OR "lay health worker*" OR "lay counsel*" OR "lay-counsel*" OR "alternative health worker*" OR "task shifting" OR "task sharing" OR "lay-delivered" OR "nonprofessional" OR "non-professional" OR "peer deliver*" OR "peer-deliver*" OR "peer volunteer*" OR "Thinking Healthy Program*" OR "scaling up" OR "scaling-up" OR "lady health worker*" OR "lady health visitor*" OR "friendship bench*" OR "barefoot therapist*" OR "barefoot doctor*" OR "community-based reproductive health agent*" OR "health extension worker*"​​ OR "accredited social health activist*" OR nonspecialist OR "non-specialist" |
| **Outcome** | **MeSH**  (MH "Depression, Postpartum") OR (MH "Mental Disorders") OR (MH "Mental Health") |
|  | **Keywords**  "mental health" OR "mental disorder*" OR depress* OR "perinatal depression" OR "postpartum depression" OR "post-partum depression" OR distress* OR psych* N2 distress* OR anxi* OR mood OR nervous* OR sad* OR uneas* OR hopeless* OR worthless* OR "baby blue*" OR tens* OR fatigue* OR tired* OR nervous* OR suicid* OR agitat* OR somati* OR somati* N2 present* OR somati* N2 symptom* OR appetite OR "mental wellness" OR sleep OR "stress reduction" OR "emotional support" |

**Global Health (via OVID)**

| **Population** | **Subject Headings**  Exp Pregnancy/ OR Exp Health Services/ OR Exp Maternity/ |
| --- | --- |
|  | **Keywords**  pregnan* OR perinat* OR prenat* OR "post-partum" OR "post partum" OR postpartum OR antenat* OR mother* OR puerper* |
| **Intervention** | **Subject Headings**  Exp Community Health Workers/ OR Volunteers/ OR Auxiliary workers/  OR Task shifting/ |
|  | **Keywords**  "community health worker*" or "community health agent*" or "lay health worker*" or "lay counsel*" or "lay-counsel*" or "alternative health worker*" or "task shifting" or "task sharing" or "lay-delivered" or "nonprofessional*" or "non-professional*" or "peer deliver*" or "peer-deliver*" or "peer volunteer*" or "Thinking Health Program*" or "scaling up" or "scaling-up" or "lady health worker*" or "lady health visitor*" or "friendship bench*" or "barefoot therapist*" or "barefoot doctor*" or "community-based reproductive health agent*" or "health extension worker*" or "accredited social health activist*" or nonspecialist* or "non-specialist*" |
| **Outcome** | **Subject Headings**  Mental Disorders/ OR Mental Health/ OR Postpartum Period/ |
|  | **Keywords**  "mental health" or "mental disorder" or depression or depressed or "perinatal depression" or "postpartum depression" or distress or psych* distress or anxi* or mood or "mood disorder*" or nervous or sad* or uneas* or hopeless* or worthless* or "baby blue*" or tens* or fatigue* or tired* or nerv* or suicid* or agitat* or somati* or "somati* present*" or "somati* symptom*" or "appetite" or "mental wellness" or "sleep" or "stress reduction" or "emotional support" |

**APA PsychINFO (via EBSCOhost)**

| **Population** | **Index terms**  DE "Mothers" OR DE "Pregnancy" OR DE "Prenatal Care OR DE "Postnatal Period" |
| --- | --- |
|  | **Keywords**  pregnan* OR perinat* OR prenat* OR "post-partum" OR "post partum" OR postpartum OR antenat* OR mother* OR puerper* |
| **Intervention** | **Index terms**  DE "Volunteers" OR DE "Peer Counseling" |
|  | **Keywords**  "community health worker*" OR "community health agent*" OR "lay health worker*" OR "lay counsel*" OR "lay-counsel*" OR "alternative health worker*" OR "task shifting" OR "task sharing" OR "lay-delivered" OR "nonprofessional" OR "non-professional" OR "peer deliver*" OR "peer-deliver*" OR "peer volunteer*" OR "Thinking Healthy Program*" OR "scaling up" OR "scaling-up" OR "lady health worker*" OR "lady health visitor*" OR "friendship bench*" OR "barefoot therapist*" OR "barefoot doctor*" OR "community-based reproductive health agent*" OR "health extension worker*"​​ OR "accredited social health activist*" OR nonspecialist OR "non-specialist" |
| **Outcome** | **Index terms**  DE "Mental Health" OR DE "Mental Disorders" OR DE "Postpartum Depression" |
|  | **Keywords**  "mental health" OR "mental disorder*" OR depress* OR "perinatal depression" OR "postpartum depression" OR "post-partum depression" OR distress* OR psych* N2 distress* OR anxi* OR mood OR nervous* OR sad* OR uneas* OR hopeless* OR worthless* OR "baby blue*" OR tens* OR fatigue* OR tired* OR nervous* OR suicid* OR agitat* OR somati* OR somati* N2 present* OR somati* N2 symptom* OR appetite OR "mental wellness" OR sleep OR "stress reduction" OR "emotional support" |

**EMBASE (via OVID)**

| **Population** | **Subject Headings**  exp pregnancy/ OR birth/ OR exp puerperium/ OR prenatal care/ OR exp maternal care/ OR maternal health services/ |
| --- | --- |
|  | **Keywords**  pregnan* or perinat* or prenat* or "post-partum" or "post partum" or postpartum or antenat* or mother* or puerper* |
| **Intervention** | **Subject Headings**  exp health auxiliary/ OR volunteer/ |
|  | **Keywords**  "community health worker*" or "community health agent*" or "lay health worker*" or "lay counsel*" or "lay-counsel*" or "alternative health worker*" or "task shifting" or "task sharing" or "lay-delivered" or "nonprofessional*" or "non-professional*" or "peer deliver*" or "peer-deliver*" or "peer volunteer*" or "Thinking Health Program*" or "scaling up" or "scaling-up" or "lady health worker*" or "lady health visitor*" or "friendship bench*" or "barefoot therapist*" or "barefoot doctor*" or "community-based reproductive health agent*" or "health extension worker*" or "accredited social health activist*" or nonspecialist* or "non-specialist*" |
| **Outcome** | **Subject Headings**  exp mental health/ OR exp postnatal depression/ OR exp mental disease/ |
|  | **Keywords**  "mental health" or "mental disorder*" or depress* or "perinatal depression" or "postpartum depression" or distress* or "psych* distress*" or anxi* or mood or "mood disorder*" or nerv* or sad or uneas* or hopeless* or worthless* or "baby blue*" or tens* or fatigue* or tired* or suicid* or agitat* or somati* or "somati* present*" or "somati* symptom*" or appetite or "mental wellness" or "sleep" or "stress reduction" or "emotional support" |
