# Supplementary material for: Peer counseling for perinatal depression in low- and middle-income countries: A scoping review
Source: Glob Ment Health (Camb). 2024 Oct 18;11:e85. doi: 10.1017/gmh.2024.73 (PMC11504931; doi:10.1017/gmh.2024.73)
Supplement: Cuncannon et al. supplementary material 2 — Cuncannon et al. supplementary material [file S2054425124000736sup002.docx]

**Peer counselling for perinatal depression in the Global South: A scoping review**

**Supplementary material**

Table 1. Types of included records

| **Type** | **Number** |
| --- | --- |
| Book chapter | 4 |
| Cohort | 7 |
| Cross-sectional | 2 |
| Meta-analysis | 1 |
| Mixed methods | 5 |
| Protocol | 6 |
| Qualitative | 13 |
| Randomized control trial | 13 |
| Report | 3 |
| Review | 2 |
| Secondary analysis | 5 |
| Systematic review +/- meta-analysis | 12 |

Table 2. Overview of included systematic reviews and meta-analyses

| **Authors** | **Countries** | **Relevant and total studies (n)** | **Purpose** | **Interventions within relevant studies** | **Major themes** |
| --- | --- | --- | --- | --- | --- |
| Chowdhary et al. (2014) | India  Pakistan | 2 (9) | Describes the content and delivery of interventions for perinatal depression by non-specialist health workers (NSHW) in low and middle income countries. Explores different interventions, strategies and techniques used to deliver the interventions, how to ensure cultural relevance, characteristics of NSHW, and challenges faced. | -*India*: Participatory women’s group  -*Pakistan*: Thinking Healthy Program (THP) | -Screening and raising awareness  -Build capacity among participants and providers  -Contextual and cultural adaptation  -Cost-effectiveness  -Provider considerations |
| Clarke et al. (2013) | India  Pakistan | 3 (11) | Focuses on interventions delivered by non-mental health specialists (NMHS) in community and primary care settings in LMICs due to a lack of mental health professionals. Aims to use insights gained to inform policy recommendations. | *Pakistan*: CBT-based  *Pakistan*: Learning Through Play programme  *India*: Participatory community groups | -Heterogeneity |
| Dennis (2013) | India | 1 (28) | Assesses the impact of psychosocial and psychological interventions in reducing the risk of perinatal depression compared with usual perinatal. Also aims to examine the effectiveness of various types of interventions such as professional versus non-professionally led and group versus individually focused, onset of intervention, and impact among women with various risk factors. | Participatory community groups | -Task-sharing |
| Fang et al. (2022) | India  Iran  Pakistan  Zimbabwe | 5 (16) | Evaluates the impact of peer support on perinatal depression and highlights qualities of effective peer support intervention. | *India*: Individual sessions  *Iran*: Peer-based telephone support  *Pakistan*: Participatory community groups  *Pakistan*: Peer-based individual and group sessions  *Zimbabwe*: Peer-support groups | -Contextual and cultural adaptation |
| Gajaria and Ravindran (2018) | India  Pakistan | 4 (18) | Focuses on interventions that target perinatal depression in LMICs. | *Pakistan* (3): THP  *India*: Participatory community groups | -Task-sharing  -Contextual and cultural adaptation  -Heterogeneity |
| Munodawafa et al. (2018) | India  Pakistan | 2 (3) | Reviews qualitative evidence of process evaluations of task sharing interventions for perinatal depression in LMICs in relation to the UK Medical Research Council framework for conducting process evaluations. | *India*: Psychoeducation, problem solving therapy  *Pakistan*: THP | -Task-sharing  -Contextual and cultural adaptation  -Development of trusting relationships  -Cascaded model  -Provider considerations |
| Padmanathan and Silva (2013) | Pakistan | 1 (21) | Summarizes task-sharing strategies aimed to reduce the treatment gap for mental disorders and discusses barriers that need to be addressed for scale-up. | CBT-based | -Contextual and cultural adaptation |
| Prina et al. (2023) | India  Gambia  Ghana | 4 (23) | Assesses the effectiveness of task-sharing psychosocial interventions as demonstrated in RCTs for preventing common mental disorders among women in the perinatal period living in LMICs. | *Ghana*: Integrated Mothers and Babies Course  *India*: Participatory community groups  *India*: Problem-based low-intensity group  *Gambia*: Community psychosocial music intervention (CHIME) | -Task-sharing  -Heterogeneity  -Contextual and cultural adaptation |
| Rahman et al. (2013) | India  Pakistan  Uganda | 5 (13) | Assesses the effectiveness of interventions that aim to improve perinatal mental health and evaluates their effect on infants and mother-infant relationships in LMICs. | *India*: Participatory community groups  *India*: Individual sessions  *Pakistan*: Learning Through Play program  *Pakistan*: THP  *Uganda:* Emotional Self-Management Group Training programme and individual sessions | -Development of trusting relationships  -Contextual and cultural adaptation |
| van Ginneken et al. (2013) | Pakistan | 1 (38) | Assesses the effectiveness of non-specialist health workers in delivering mental health interventions in LMICs. | THP | -Task-sharing |
| van Ginneken et al. (2021) | India  Pakistan  Zimbabwe | 4 (95) | Evaluates the effectiveness of primary-worker led mental health interventions in LMICs, including interventions that target perinatal depression. | *India:* THP  *Pakistan:* THP  *Pakistan:* THP  *Zimbabwe:* PST | -Task-sharing  -Heterogeneity |
| Waqas et al. (2023) | No country disaggregation | No country disaggregation | Assesses the effectiveness of CBT-based interventions as demonstrated in RCTs for prevention and treatment of perinatal depression, explores the settings of highest effectiveness, explores individual- and intervention-level factors in prognosis, and components of CBT interventions. | CBT-based | -Task-sharing  -Development of trusting relationships  -Contextual and cultural adaptation |
| Zhu et al. (2022) | Ethiopia  India  Indonesia  Malawi  Nigeria  Pakistan  Uganda | 8 (17) | Uses the Consolidated Framework for Implementation Research to identify factors that facilitate the implementation of task-sharing interventions aimed to address perinatal depression in LMICs. | *Ethiopia*: Psychological interventions  *Ethiopia*: Program for Improving Mental health care (PRIME) and The Emerging mental health system (Emerald)  *India*: THP  *Indonesia*: Seeking and accepting treatment  *Malawi*: THP  *Nigeria*: Interventions using the WHO mhGAP intervention guide (mhGAP-IG)  *Pakistan*: THP  *Pakistan*: THP  *Uganda*: PRIME | -Screening and raising awareness  -Contextual and cultural adaptation  -Appropriate technology  -Development of trusting relationships |

Table 3. Geographies of included interventional studies

| **Country** | **Number^a^** |
| --- | --- |
| Afghanistan | 1 |
| Bangladesh | 3 |
| Ghana | 1 |
| India | 11 |
| Indonesia | 1 |
| Iran | 1 |
| Kenya | 6 |
| Malawi | 1 |
| Nepal | 1 |
| Pakistan | 27 |
| Swaziland | 1 |
| Uganda | 1 |
| Vietnam | 1 |
| Zimbabwe | 1 |

^a^Reflects studies spanning multiple countries

Table 4. Primary programs of included interventional studies

| **Modality** | **Number** |
| --- | --- |
| Cognitive behavioral therapy (CBT) | 2 |
| Happy Mother, Healthy Baby | 2 |
| Happy Mother, Healthy Child in Ten Steps | 1 |
| Integrated Mothers and Babies Course & Early Childhood Development (iMBC/ECD) | 2 |
| Interpersonal therapy (IPT) | 4 |
| Learning Through Play Plus (LTP+) | 1 |
| Parenting, integrated (cognitive, behavioural, interpersonal, and simulation) | 1 |
| Peer-led participatory groups and community mobilization | 1 |
| Problem-solving therapy (PST) | 1 |
| Siphilile (“We are Healthy”) | 1 |
| Thinking Healthy Programme (THP) | 11 |
| Thinking Healthy Programme peer-delivered (THPP) | 16 |

Additional modalities included group problem-solving therapy (Chibanda *et al.* 2014; George *et al.* 2020), a group CBT-based Integrated Mothers and Babies Course and Early Childhood Development intervention (Kim *et al.* 2021), telephone support (Shamshiri Milani *et al.* 2015), peer-led participatory groups and community mobilization (Tripathy *et al.* 2010), (Yeasmin *et al.* 2021), and interpersonal therapy (Yator *et al.* 2021).

Table 5. Overview of articles examining THPP

| **Author and year** | **Countries** | **Aims/purpose** | **Methods** | **Key findings** |
| --- | --- | --- | --- | --- |
| Ahmad et al. (2020) | Pakistan | Report on development of an Implementation Strength Index for THPP. | Embedded within Sikander et al.’s (2019) cluster RCT. | Peer volunteers demonstrated good scores on implementation indices; however, there was no significant association between implementation indices and PHQ-9 scores. Other constructs may underlie the association between THPP intervention and PHQ-9 scores. |
| Atif et al. (2016) | Pakistan | Exploration of stakeholders' experiences of THPP (i.e., barriers and facilitators to the acceptability of peer volunteers) prior to implementation. | Qualitative study embedded within the pilot phase of THPP: interviews and focus group discussions. | Facilitators of peer volunteers’ motivation included personal gains, family and community endorsement, and good training and supervision. Barriers to peer volunteers’ motivation included lack of engagement of mothers, resistance from mothers' families. Facilitators to community acceptability included high level of need, desirable peer volunteer characteristics, linkages with local primary health care system, and the intervention being perceived positively. Barriers to community acceptability included stigma regarding mental illness and societal and cultural barriers. |
| Atif et al. (2017) | India  Pakistan | Adaptation and feasibility (field) testing of THP for delivery by peers in Goa and rural Rawalpindi. | Qualitative study: focus groups and field testing. | Most mothers reported some improvement, including better coping abilities and motivation to take action and spend more quality time with their children. Peers were perceived to have good social and cultural understanding and relatability. Peers saw the opportunity as a way to learn new skills and enhance employment opportunities. There was an expressed desire for monetary incentives for peers in Goa, in contrast to rural Rawalpindi where status and goodwill were perceived as sufficient. |
| Atif, Nisar, et al. (2019) | Pakistan | Evaluation of cascaded model of training and supervision to sustain delivery of THP in rural Pakistan. | Mixed methods (Quality and Competency Checklist and focus groups) embedded within Sikander et al.’s (2019) cluster RCT. | The cascaded model was effective in guiding training and supervision of local peer volunteers who demonstrated competency to deliver THP. Most peer volunteers showed sustained or improved competence 6, 12, and 24 months after training. Facilitators of training and supervision included trainer relatability, perceived usefulness, role play and experiential strategies, and linkages with primary health system. Barriers to training and supervision included lack of refresher trainings, challenges with Skype, and competing responsibilities and household commitments. |
| Atif, Bibi, et al. (2019) | Pakistan | Preliminary evaluation of THPP in rural Rawalpindi 5 years after initiation. | Mixed methods (Quality and Competency Checklist and interviews) | After 5 years, 70% of peer volunteers in the original cohort continued to be part and all achieved satisfactory competence. Factors contributing to sustained motivation included altruism, enhanced social standing, improved personal mental health, and the possibility for other employment opportunities. Challenges included demotivation associated with uncertainty about the programme's future, increased desire for financial incentives, and resistance to ongoing care from some families. |
| Atif et al. (2022) | Pakistan | Development of technology-assisted peer-delivered Thinking Healthy Programme in rural Rawalpindi. | Usability testing study with a human-centred design approach | Challenges were encountered with engagement; strategies to enhance engagement included a narrative approach for key messages with vignettes as well as family involvement. The peer and App are positioned as ‘co-therapists.’ The App can be used offline once downloaded. The next phase will be an RCT to compare technology-assisted peer delivered with conventional delivery. |
| Fuhr et al. (2019) | India | Assessment of the effectiveness and cost-effectiveness of THPP in Goa, India. | RCT: THPP and enhanced usual care (EUC) as intervention compared to EUC as control. | Higher prevalence of remission at 6 months’ time after birth in the intervention (THPP plus EUC) group compared with the control (EUC only) group and no significant difference in symptom severity at 6 months’ time between intervention and control group. Reductions in symptom severity were greatest within the first 3 months after childbirth, which may be related to front-loading of sessions and intervention content. THPP significantly improved women’s perceived social support and reduced disability during the first 6 months and 3 months, respectively, after childbirth. |
| Leocata et al. (2021a) | India  Kenya | Exploration and contrasting of material technologies in lay-counsellor interventions in India and Kenya. | Ethnography based on fieldwork with THPP in India and Tuko Pamoja in Kenya. | Material technologies of care can both facilitate relational engagement and constrain care. Historical dynamics with the lay counselling role persist in that intervention materials suggest utilization of lay counsellors’ experience and expertise, but tensions like manualization, protocolization, and evaluation remain. |
| Leocata et al. (2021b) | India | Examination of how caregiving is experienced by peer counsellors in THPP. | Ethnography based on fieldwork during THPP RCT in India. | Three primary themes regarding caregiving and end-of-care as experienced by the sakhis: caregiving as a relational exchange and reciprocated process, memories of care and moral resonance, and awareness surrounding disengagement of care. |
| Maselko et al. (2020) | Pakistan | Evaluation of the effects of the Thinking Healthy Program, Peer-delivered Plus (THPP+) on maternal depression and child development at 36 months. | Cluster RCT of THPP+ (peer-support, behavioural activation, problem solving, and developmental activities for children up to 36 months postnatal) | No significant outcome differences, as measured by PHQ-9 scores and Strengths and Difficulties Questionnaire, between intervention and enhanced usual care arms. ~40% of women did not complete the entire THPP+ intervention. Peers’ competence levels declined over time, particularly after 12 months’ time. |
| Ng’oma et al. (2022) | Malawi | Exploration of cultural adaptation and appropriateness of THPP in Lilongwe, Malawi. | Qualitative exploratory design including focus group discussions, video recordings of theatre role plays, and content analysis. | Adapted THPP was deemed appropriate and beneficial. Recommendations included revision of illustrations for clarity, cultural adaptation of stories and idioms, and modification to the number and duration of sessions. |
| Rahman et al. (2021) | India  Kenya  Vietnam  Pakistan | Examines the impact of THP on global health policy and practice, with a particular focus on lessons learned from implementing THP in different countries and in the context of the COVID-19 pandemic. | Review of THP implementations across five years and four countries. | Highlighted are the effectiveness of peer-delivery in India, cross-cultural and health system transferability through the translation and adaptation of THP in Vietnam, technology for training and supervising in Pakistan, and mobile phone delivery in Kenya. Factors contributing to policy uptake suggested include cost effectiveness, feasibility in low-resource contexts, cross-cultural transferability, ability to integrate into existing maternal and child health programs, ability to integrate into task-sharing and cascaded models, and potential for technology to scale and expand training and program delivery. |
| Sikander et al., 2015 | India  Pakistan | Report on a trial protocol for THPP: cluster trial in Rawalpindi and an individually randomized trial in Goa | Trial protocol for THPP with a comparator group receiving EUC. | See Fuhr et al. (2019) for results in India and Sikander et al. (2019) for results in Pakistan. |
| Sikander et al., 2019 | Pakistan | Assessment of the effectiveness and cost-effectiveness of THPP in Rawalpindi, Pakistan. | RCT: THPP+EUC as intervention compared to EUC as control. | Severity of depression (assessed by PHQ-9 scores) and prevalence of remission did not significantly differ between groups at 6 months. Women in the intervention arm were more likely to show remission at 3 months as well as recovery. |
| Singla et al. (2021) | India  Pakistan | Explores the role of mediators (patient-reported activation, social support, and mother-child attachment) within THPP RCTs. | Multiple mediation analysis. | Two of three variables (patient activation and social support) mediated the effects of THPP on depression outcomes at 6 months. THPP did not influence mother-child reported attachment. |
| Vanobberghen et al. (2020) | India  Pakistan | Pooled analysis of THPP-India and THPP-Pakistan. | Pooled analysis. | Participants in intervention arm had lower symptom severity (assessed by PHQ-9 scores)and odds of remission. Intervention effect was greater among primiparious women and those with shorter depression chronicity. |

**References**

Chibanda, D, Shetty, AK, Tshimanga, M, Woelk, G, Stranix-Chibanda, L, and Rusakaniko, S (2014) Group problem-solving therapy for postnatal depression among HIV-positive and HIV-negative mothers in Zimbabwe. *Journal of the International Association of Providers of AIDS Care*, **13**(4), 335–341. doi:10.1177/2325957413495564.

George, C, Kumar, A, V, and Girish, N (2020) Effectiveness of a group intervention led by lay health workers in reducing the incidence of postpartum depression in South India. *Asian Journal of Psychiatry*, **47**. doi:10.1016/j.ajp.2019.101864.

Kim, ET, Opiyo, T, Acayo, PS, … Baumgartner, JN (2021) Effect of a lay counselor delivered integrated maternal mental health and early childhood development group-based intervention in Siaya County, Kenya: A quasi-experimental longitudinal study. *Journal of Affective Disorders*, **292**, 284–294. doi:10.1016/j.jad.2021.06.002.

Shamshiri Milani, H, Azargashb, E, Beyraghi, N, Defaie, S, and Asbaghi, T (2015) Effect of Telephone-Based Support on Postpartum Depression: A Randomized Controlled Trial. *International Journal of Fertility & Sterility*, **9**(2), 247–253. doi:10.22074/ijfs.2015.4246.

Tripathy, P, Nair, N, Barnett, S, … Costello, A (2010) Effect of a participatory intervention with women’s groups on birth outcomes and maternal depression in Jharkhand and Orissa, India: a cluster-randomised controlled trial. *The Lancet*, **375**(9721), 1182–1192. doi:10.1016/S0140-6736(09)62042-0.

Yator, O, John-Stewart, G, Khasakhala, L, and Kumar, M (2021) Preliminary Effectiveness of Group Interpersonal Psychotherapy for Young Kenyan Mothers With HIV and Depression: A Pilot Trial. *American Journal of Psychotherapy*, appipsychotherapy20200050. doi:10.1176/appi.psychotherapy.20200050.

Yeasmin, F, Winch, PJ, Hwang, ST, … Rahman, M (2021) Exploration of Attendance, Active Participation, and Behavior Change in a Group-Based Responsive Stimulation, Maternal and Child Health, and Nutrition Intervention. *The American Journal of Tropical Medicine and Hygiene*, **104**(4), 1586–1595. doi:10.4269/ajtmh.20-0991.
