# Supplementary material for: Peer counseling for perinatal depression in low- and middle-income countries: A scoping review
Source: Glob Ment Health (Camb). 2024 Oct 18;11:e85. doi: 10.1017/gmh.2024.73 (PMC11504931; doi:10.1017/gmh.2024.73)
Supplement: Peer review history

# Editor report: Recommendation: Peer counseling for perinatal depression in low- and middle-income countries: A scoping review — R0/PR2

**DOI:** 10.1017/gmh.2024.73.pr2

**Affiliations:** Fudan University, China

No accompanying comment.

# Editor report: Decision: Peer counseling for perinatal depression in low- and middle-income countries: A scoping review — R0/PR3

**DOI:** 10.1017/gmh.2024.73.pr3

**Affiliations:** London School of Hygiene & Tropical Medicine, United Kingdom of Great Britain and Northern Ireland

No accompanying comment.

# Editor report: Recommendation: Peer counseling for perinatal depression in low- and middle-income countries: A scoping review — R1/PR5

**DOI:** 10.1017/gmh.2024.73.pr5

**Affiliations:** Fudan University, China

No accompanying comment.

# Editor report: Decision: Peer counseling for perinatal depression in low- and middle-income countries: A scoping review — R1/PR6

**DOI:** 10.1017/gmh.2024.73.pr6

**Affiliations:** London School of Hygiene & Tropical Medicine, United Kingdom of Great Britain and Northern Ireland

No accompanying comment.
